# Shared mechanisms in stemness and carcinogenesis: lessons from oncogenic viruses

**DOI:** 10.3389/fcimb.2013.00066

**Published:** 2013-12-25

**Authors:** Demetris Iacovides, Stella Michael, Charis Achilleos, Katerina Strati

**Affiliations:** Department of Biological Sciences, University of CyprusNicosia, Cyprus

**Keywords:** cancer, stemness, reprogramming, HBV, HCV, HPV, EBV, KSHV

## Abstract

A rise in technologies for epigenetic reprogramming of cells to pluripotency, highlights the potential of understanding and manipulating cellular plasticity in unprecedented ways. Increasing evidence points to shared mechanisms between cellular reprogramming and the carcinogenic process, with the emerging possibility to harness these parallels in future therapeutics. In this review, we present a synopsis of recent work from oncogenic viruses which contributes to this body of knowledge, establishing a nexus between infection, cancer, and stemness.

## Introduction

Long-standing observations have noted a number of parallels between the homeostasis of cancer cells and that of stem cells. A complicated picture includes the involvement of tissue stem cells as the cells-of-origin for some cancers, a stem cell compartment thought to maintain most tumors [commonly known as cancer stem cells (CSCs)], as well as more recent concepts of differentiated cells being reprogrammed back to pluripotency during the carcinogenic process (Lapouge et al., 2011; Friedmann-Morvinski et al., 2012). Several publications have shown that classic tumor suppressors such as p53 and pRb have emerging roles in the regulation of stemness (Conklin and Sage, 2009; Bonizzi et al., 2012). In addition to that, genes generally known for their key roles in stem cell biology, for example Nanog, appear to be deregulated in a number of cancers (Zhang et al., 2012; Lu et al., 2013). In the cutting edge field of reprogramming cells to pluripotency, key players in tumor suppression have been implicated in crucial roadblocks to the reprogramming process. While there is still a lot to be understood, it has been proposed that understanding the complicated relationship between stemness and cancer may hold the key to more successful future therapies; for example targeting cancer stem cells may reduce the possibility of future cancer recurrence.

Virally-induced cancers, thought to account for about 20% of the global cancer incidence, have long been studied to enable better understanding of the clinical manifestation of the disease as well as for their value as models of carcinogenesis overall (Farrell, 2002). Such cancers are attributed mainly to Hepatitis B Virus (HBV), Hepatitis C Virus (HCV), Human Papilloma Virus (HPV), Epstein-Barr Virus (EBV), Kaposi's Sarcoma-associated Herpes virus (KSHV), Human T-cell Leukemia Virus-1 (HTLV-1), and more recently, Merkel Cell Polyoma Virus (MCPyV) (Samanta et al., 2003; Bonilla Guerrero and Roberts, 2005; Bajaj et al., 2007; Schiffman et al., 2007; Saha et al., 2010; Jeong et al., 2012; Amber et al., 2013; Cook et al., 2013). These viruses encode proteins shown to impinge on various cellular processes including cell cycle regulation, apoptosis, cell signaling, transcriptional regulation, and epigenetic regulation, resulting in carcinogenesis (Saha et al., 2010). We present here evidence which implicates oncogenic viruses in the regulation of pluripotency at various levels. We argue that virus-associated cancers can serve as models to understand the general link between cancer and stemness, as well as the distinct role that infection plays in these cases. It should be noted that other types of infectious agents, most notably the leprosy bacterium and Helicobacter pylori, have also been shown to modulate stemness-associated processes and pathways in host cells, raising the possibility that strategies involving the manipulation of cellular stemness may serve as evolutionary advantages to pathogens (Fujii et al., 2012; Wegner, 2013). Here, we review the available evidence for regulation of stemness by oncogenic viruses with particular emphasis on results coming from *in vivo* model systems. We also propose key questions that remain to be addressed.

## Interaction of oncogenic viruses with tissue stem cells

Tissue stem cells and committed tissue progenitor cells destined for terminal differentiation are target cells of several oncogenic viruses. While no known oncogenic virus displays exclusive tropism for such specific cell populations, infection of either a stem or progenitor population may provide the opportunity of a longer-lived cellular reservoir for viral replication. In addition, infection of these cells might in some cases enable viruses to evade the immune system, since tissue progenitor/stem cells might be immune privileged (Di Trapani et al., 2013), even though this notion is still controversial (Tseng et al., 2010).

Gammaherpesviruses, including KSHV and its murine cousin MHV68 and EBV infect primarily resting mature B cells. However, these cells are short-lived and non-proliferating, which points to the possibility that herpesviruses may also be able to infect a progenitor, stem cell-like population of B cells, which normally gives rise to mature B cells, in order to ensure continuous viral genome propagation and viral latency maintenance. Indeed, there is some evidence that both human and murine gammaherpesviruses infect hematopoetic progenitor cells. KSHV has been detected in immature hematopoetic cells in the bone marrow of transplant recipients (Luppi et al., 2000; Lapouge et al., 2011) and in hematopoietic progenitor cells in Kaposi's sarcoma patients (Henry et al., 1999; Friedmann-Morvinski et al., 2012), whereas MHV68 was detected in immature splenic B cells in the mouse (Marques et al., 2003; Collins et al., 2009). Moreover, KSHV-infected human hematopoietic progenitor stem cells gave rise to KSHV-infected mature human B-cells and monocytes when transplanted in NOD/SCID mice (Wu et al., 2006). Coleman et al. examined developing B cell infection by MHV68, a model for gammaherpesviruses, in a fully immunocompetent mouse host. They showed that this virus establishes long-term latency in immature B cells in the bone marrow as well as in transitional B cells in the spleen (Coleman et al., 2010). Since these self-renewing stem cell populations of developing B cells give rise to mature resting B cells, the authors speculate that infection of these cell populations by herpesviruses might play a key role in the maintenance of lifelong infection in the host.

Even though the direct involvement of Human Cytomegalovirus (HCMV) in tumor initiation is still not well-documented, a variety of malignancies have been associated with HCMV infections and persistence but the association is more widely accepted for malignant gliomas (Harkins et al., 2002; Samanta et al., 2003; Soderberg-Naucler, 2006; Michaelis et al., 2009). In normal brain tissue, HCMV appears to primarily target cells in the subventricular zone (SVZ) of the brain (Perlman and Argyle, 1992; Fritschy et al., 1996; Odeberg et al., 2007), which is the source of local stem cells and progenitor cells within this organ (Seri et al., 2006). Differentiation of neural precursors into mature neurons seems to reduce susceptibility to HCMV infection (Lokensgard et al., 1999; Cheeran et al., 2005) and activation of PDGFR alpha (essential to the self-renewal potential of neural stem cells) (Kofman et al., 2011) by HCMV is necessary for successful infection (Soroceanu et al., 2008). These results further support the possibility that the primary cell reservoir for HCMV, at least in the brain, is the stem cell compartment (Dziurzynski et al., 2012), and that infection of HCMV of this cell population might be a way for the virus to successfully establish lifelong latency in the host.

HPVs are strongly associated with a number of malignancies, most notably cervical carcinoma (CC). Several studies have proposed the existence of multiple HPV target cells within the host epithelium. There is increasing support for the hypothesis that stem cells of the transformation zone (TZ) of the cervical epithelium are the primary site of persistent HPV infection (Lopez et al., 2012). Given the anatomical observation that a lot of cervical cancers are derived from the TZ, a connection between infection of tissue stem cells and eventual carcinogenesis has been proposed. The long latency period between infection with HPV and development of cervical dysplasias supports the hypothesis that these cells can be targets of HPV infection and serve as a vehicle for long-term established viral latency in the cervix. Using laser capture microdissection in a rabbit oral papillomavirus (ROPV) model system, Maglennon et al. (2011) showed that ROPV indeed persists in a latent state, even after immune-mediated regression of induced papillomas, and that the site of latency is a subset of basal epithelial cells which the authors propose are the epithelial stem cells. It should be noted that expression of papillomavirus genes in stem cells has been shown to modulate their behavior *in vivo* and may be associated with ensuing carcinogenesis. In a study using mice transgenic for the HPV16 oncogenes our group showed that expression of viral oncogenes in label-retaining epithelial stem cells caused aberrant mobilization (Michael et al., 2013). In a related study, using animals expressing the entire HPV16 viral genome in all basal cells of stratified epithelia, skin cancers were shown to derive from tissue stem cells (da Silva-Diz et al., 2013).

## Viruses giving rise to cancer stem cells

CSCs are cells within a tumor that possess stem cell properties, namely the ability to self-renew and give rise to progeny destined for differentiation to regenerate tumor cell diversity. Though genetic changes or oncogenic infection of an undifferentiated cell is usually thought to give rise to tumor initiating cells, tumors have been shown to originate from differentiated cells as well (Friedmann-Morvinski et al., 2012). It has been suggested that cellular reprogramming mediated by oncogenic viruses may promote the formation of tumor initiating cells or CSCs. The term “tumor initiating cells,” strictly referring to the initial cells from which a tumorigenic transformation occurs, is used interchangeably in most cases, describing the ability of CSCs to fully regenerate, or “reinitiate” the tumor.

Several reports have implicated oncogenic viruses in the generation of CSCs. Arzumanyan et al. recently showed that HBV might induce initiation of hepatocellular carcinomas (HCC) by activating cellular factors that promote stemness (Arzumanyan et al., 2011). HBV encoded X antigen (HBVx), important in the viral life cycle as well as carcinogenesis, was shown to activate stemness associated factors Oct-4, Nanog, Klf4, beta catenin, and EpCAM *in vitro*. In addition, this protein was shown to induce cell migration, sphere formation, and growth in soft agar, all phenotypic characteristics of CSCs. These results were confirmed in liver biopsies obtained from HCC patients, since the above stemness associated markers were observed in the majority of HBV associated HCCs (Arzumanyan et al., 2011). Interestingly, microarray data from HBV-associated HCC showed that miR-181, recently found to contribute to tumorigenesis (Agami, 2010), was over-expressed in hematopoietic stem cells (HSCs) and CSCs, and was also found to be upregulated in HBx-expressing cells and HBx-positive liver biopsies (Arzumanyan et al., 2011) suggesting that this micro-RNA might be involved in stemness or CSCs induction and maintenance in HBV-associated HCCs.

The HCV has also been implicated in induction of CSCs. Machida et al. isolated tumor initiating stem-like cells from transgenic mice expressing HCV core, as well as from patients with HCC, and showed that the Tlr4-Nanog pathway was upregulated in these cells and was necessary for their tumorigenic properties (Machida et al., 2009, 2012). Nanog, a stem/progenitor cell marker was further shown to be upregulated through activation of the TLR4 pathway by NS5A, a non-structural protein encoded by HCV (Machida et al., 2012). Furthermore, a study by Ali et al. showed that infection of cultured hepatic cells with an HCV subgenomic replicon resulted in acquisition of CSC characteristics, including expression of Lgr5, c-myc, and DCAMKL-1 (Ali et al., 2011). A DCAMKL-1 enriched cell population was subsequently shown to form tumors with expression of proteins associated with metastatic potential in athymic nude mice. Importantly, removing the HCV replicon from these cells dramatically reduced expression of the stem cell-associated markers. The results correlated well with analysis of liver biopsies from HCV-infected patients, further highlighting the possibility that HCV promotes a CSC-like phenotype *in vivo*.

Several studies have suggested the possibility that EBV might exert its tumorigenic properties at least in part by giving rise to CSCs within the infected tissue. In an important study, Kong et al. investigated the role of EBV LMP2A protein in CSC modulation in nasopharyngeal carcinoma (NPC) cells, and showed that expression of this protein induced cell invasion and epithelial-mesenchymal transformation (EMT) (Kong et al., 2010). Overexpression of LMP2A was found to enrich stem cell like cells within the NPC tumor cell population, and increased the number of cells that were capable of re-establishing tumors in nude mice (Kong et al., 2010). These results were subsequently confirmed in NPC patient biopsies, further suggesting that a possible mechanism of tumorigenesis in EBV-infected tissues is the modulation of the tissue stem cell compartment and the induction of tumor initiating cancer stem cells. A subsequent study showed that, similar to LMP2A, EBV encoded LMP1 latent membrane protein also stimulated EMT, induced a CSC/CPC-like phenotype and enhanced the self-renewal potential in nasopharyngeal epithelial cell lines, further supporting EBV involvement in modulation of cellular plasticity and induction of CSC cellular phenotypes (Kondo et al., 2011). This notion is also highlighted by a more recent study (Lun et al., 2012), which showed up-regulation of multiple stem cell markers in an EBV-positive NPC cell line with increased tumorigenic potential and high resistance to chemotherapy. Finally, a recent study by Port et al. demonstrated that NPC is frequently associated with deregulation of the Hedgehog (HH) pathway, a pathway that is associated with stem cell maintenance. In an *in vitro* model of NPC, the authors showed that EBV activates the HH pathway through induction of the SHH ligand, which leads to increased expression of stemness-associated genes and induction of stem cell phenotypes in these cells (Port et al., 2013).

The long length of papillomavirus infection usually preceding malignant pathologies has been proposed to relate to latency of viral infection in tissue stem cells. Infected tissue stem cells may serve as tumor initiating or CSC in HPV-induced CCs. In support of this hypothesis, a study showed that the invasive and metastatic potential of cervical squamous cell carcinoma (CSCC) was correlated with cancer stem cell-associated genes, and supported the idea that high-risk HPV might induce CSC phenotypes in the TZ of the cervical epithelium (Liu et al., 2010). In addition, expression of HPV E6 and E7 viral oncogenes was shown to induce epigenetic reprogramming in human keratinocytes, through modulation of chromatin structure and global methylation/acetylation events involving cellular factors that have significant role in tumorigenesis and stemness. For example, Hyland et al. showed that E6/E7-expressing primary human foreskin keratinocytes have elevated levels of the EZH2 methyltransferase and the KDM6A demethylase, which results in a reduction of global H3K27 trimethylation and upregulation of downstream targeted HOX genes (Hyland et al., 2011). Reduction in trimethylation of H3K27 associated with elevated EZH2 was also demonstrated in high-grade squamous cervical intraepithelial lesions. In a related study, McLaughlin et al. demonstrated that repressive H3K27 trimethylation was reduced in HPV-positive cervical lesions, and that this was a result of E7-mediated induction of KDM6A and KDM6B demethylases, which subsequently lead to significantly higher expression of homeobox genes (McLaughlin-Drubin et al., 2011). These findings support the possibility that HPV-induced epigenetic reprogramming is important in viral oncogenesis, and further highlight the commonalities between stemness and carcinogenesis, at least in the context of the oncogenic virus life cycle. Further research is needed to fully understand whether HPV-associated cancers are related to cellular reprogramming of infected tissue stem cells or more differentiated cells. The impact of such reprogramming on the viral life cycle also remains unknown.

## Pathways targeted by oncogenic viruses are associated with stemness

A number of reports have shown that classic tumor suppressors and their pathways, notably p53 and pRb, which are long known to be targets of oncogenic viruses (Felsani et al., 2006; Levine, 2009), have important roles in modulation of stemness.

The p53/ARF pathway is a well-established stemness repressor and cells in which this pathway is inactivated can be more efficiently reprogrammed to pluripotency (Hanna et al., 2009; Hong et al., 2009; Kawamura et al., 2009; Li et al., 2009; Marion et al., 2009; Utikal et al., 2009). p53 was also recently found to induce miR-34a and miR-145, which negatively regulate stemness-associated factors (Xu et al., 2009; Jain et al., 2012). More recently, two separate reports further highlighted the importance of p53 in stem cell biology. Chiche et al. showed that somatic loss of p53 resulted in higher numbers of stem/progenitor cells in mammary epithelium (Chiche et al., 2013). Sato et al. reported that p53 activation promoted proteosome-dependent degradation of Nanog and differentiation of glioma stem cells (Sato et al., 2013). It is therefore possible that p53 inactivation, a common strategy of oncogenic viruses, may contribute positively to the viral life cycle in a way additional to the proposed viral escape of apoptosis of infected cells.

The retinoblastoma tumor suppressor (pRb) is another major target of oncogenic viruses, since inhibition of Rb liberates the E2F transcription factor, which stimulates entry of the cell into the cell cycle, thus favoring viral replication. Increasing evidence has implicated this pathway in stemness modulation, initially in plants (Ebel et al., 2004; Wildwater et al., 2005) and subsequently in animals (Liu et al., 2009). Accumulating evidence reinforces the role of pRb in stem cell homeostasis (Conklin and Sage, 2009). The pRb pathway was shown to have a critical role as a roadblock in the reprogramming of human fibroblasts to iPSCs, as well as cell fate determination, as elegantly shown by Calo et al. (2010). Conceivably then, like p53 inactivation, the inactivation of pRb could promote cellular plasticity and stemness, which in turn would confer an ideal niche for virus persistence and latency.

There is mounting evidence supporting the recently suggested notion that tumor suppressor pathways, traditionally key targets of oncogenic viruses, might play a significant role in cellular plasticity and modulation of stemness. Even cellular factors activated by genetic events in virally-induced cancers such as c-myc in Burkitt's lymphoma, have well-described involvement in cancer as well as stemness (Dang, 2012; Buganim et al., 2013). Therefore, it is also not surprising that factors traditionally involved in stemness and cellular plasticity are increasingly being identified as targets of oncogenic viruses. Indeed, HCV, HBV, and EBV have been shown to regulate a number of pluripotency and stem cell-associated factors (Ruf et al., 1999; Machida et al., 2009; Ali et al., 2011; Lun et al., 2012). In addition, telomerase activation and telomere maintenance are important in both cancer and stemness, and it is therefore not surprising that oncogenic viruses evolved to regulate these processes. Most, if not all, tumor viruses, including the oncogenic retrovirus HTLV-1, induce transcriptional activation of telomerase (Kuhlmann et al., 2007; Bellon and Nicot, 2008), and EBV and HPV are also known to regulate telomerase post-transcriptionally.

## Discussion

**Figure 1:**
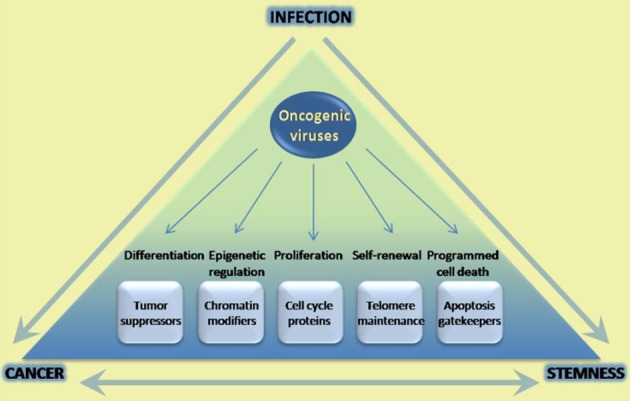
**Infection with oncogenic viruses highlights parallels in cancer and stem cell biology.** Oncogenic viruses modulate a variety of cellular pathways with parallel roles in the carcinogenic process and stem cell homeostasis. The parallels between these two processes have been extensively documented, and increasingly well-understood in terms of being able to reprogram cell state. However little has been done in the way of uncovering potential roles of these pathways in infection success. Increasing understanding of common pathways modulated may yield better tools to prevent and treat infection, as well as ensuing carcinogenesis.

Oncogenic viruses cause cancer after long-term infection of their natural niche. These viruses interfere with signaling pathways that are important in a number of major cellular processes including cell proliferation and cell division, apoptosis, and cell differentiation. Accumulating evidence suggests that oncogenic viruses may also manipulate cellular stemness in various ways. Stem cells or progenitor cells are targets of infection and normal cell homeostasis is disrupted as a result. Moreover, pathways that are traditionally associated with self-renewal and lineage-commitment have been shown to be transcriptionally regulated by viral oncoproteins. Regulation of such pathways, and of oncogenic pathways now understood to play key roles in stemness, may lead to cellular reprogramming. Whether regulation of stemness is necessary for ensuing carcinogenesis, or whether it has any impact on the viral life cycle, has not been conclusively addressed. However, it is conceivable that infection of tissue stem cells might positively affect the viral life cycle, especially in terms of establishing a successful chronic infection (Figure 1). It should also be noted that regulation of innate immunity and inflammation, also known to be linked to carcinogenesis, is now beginning to be linked to stemness as well (e.g., TLR4-Nanog, TLR3) (Machida et al., 2009; Lee et al., 2012). Additional studies are necessary in order to fully investigate this notion, especially in the context of *in vivo* infection models. As we continue to explore the parallels between cellular stemness and the carcinogenic process, oncogenic viruses continue to serve as excellent paradigms with plenty to teach.

### Conflict of interest statement

The authors declare that the research was conducted in the absence of any commercial or financial relationships that could be construed as a potential conflict of interest.
